# Does Motility‐Restricting Fibrosis Influence Dispersal? An Experiment in Nature With Threespine Stickleback

**DOI:** 10.1002/ece3.70697

**Published:** 2024-12-12

**Authors:** Alexis M. Heckley, Daniel I. Bolnick, Francis Dinh, Andrew P. Hendry, Natalie C. Steinel

**Affiliations:** ^1^ Department of Biology McGill University Quebec Canada; ^2^ Department of Ecology Evolutionary Biology University of Connecticut Storrs Connecticut USA; ^3^ Department of Biological Sciences University of Massachusetts Lowell Lowell Massachusetts USA; ^4^ Center for Pathogen Research and Training University of Massachusetts Lowell Lowell Massachusetts USA

**Keywords:** fibrosis, immunity, locomotion, movement, parasitism, *Schistocephalus*

## Abstract

Dispersal can affect individual‐level fitness and population‐level ecological and evolutionary processes. Factors that affect dispersal could therefore have important eco‐evolutionary implications. Here, we investigated the extent to which an inflammation and tissue repair response—peritoneal fibrosis—which is known to restrict movement, could influence dispersal by conducting a mark‐recapture experiment in a lake in Alaska with threespine stickleback (*Gasterosteus aculatus)*. A subset of captured stickleback were injected with aluminium phosphate to experimentally induce fibrosis (‘treatment group’), and another subset were injected with saline or received no injection—both of which do not induce fibrosis (‘control group’). We released all fish at one introduction point and re‐sampled stickleback throughout the lake for 8 days. We recaptured 123 individuals (*n* = 47 fibrosis treatment; *n* = 76 control) and dissected them to determine fibrosis levels. Overall, fibrosis did not affect dispersal. Some compelling (but not statistically significant) trends suggest that early‐stage inflammation may affect dispersal, providing opportunities for future work. By showing that effects on dispersal are not important side effects of fibrosis, these findings improve our understanding of the ecological implications of immune responses.

## Graphical abstract

Factors that affect dispersal can have important eco‐evolutionary implications. Here, we show that an inflammation and tissue repair response—peritoneal fibrosis—does not influence threespine stickleback dispersal in a lake in Alaska.

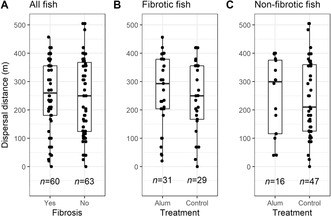

## 1 Introduction

Dispersal, or the movement of organisms with potential for gene flow, can have implications for individual fitness (Ronce 2007). Dispersal can have fitness benefits in cases where organisms disperse to avoid inbreeding, competition, or suboptimal environments (Bonte et al. 2012). Yet dispersal can be detrimental to individual fitness as it is energetically costly, does not guarantee access to superior habitats and can increase exposure to predators or parasites (Bonte et al. 2012). Any factors that influence dispersal could therefore have important eco‐evolutionary implications.

Immune responses represent one factor that can influence host dispersal (Brown and Shine 2014; e.g. Møller, Martín‐Vivaldi, and Soler 2004; Suhonen, Honkavaara, and Rantala 2010). Following immune activation, dispersal could increase due to individuals seeking out resources or environments (e.g. behavioural fever; Rakus, Ronsmans, and Vanderplasschen 2017) or because some immune responses are associated with hormones that correspond to increased dispersal (e.g. testosterone can be associated with both dispersal and aspects of immune functioning in some mammals; Holekamp and Smale 1998; Muehlenbein and Bribiescas 2005). Alternatively, dispersal could decrease due to lethargy associated with sickness behaviour (Tizard 2008) or physical changes to host structures or tissues in response to infection that affect motility. As an example of the latter, fibrosis is an early immunological (inflammatory) and tissue repair response characterised by the development of collagenous tissue in the host body cavity that can result in increased tissue stiffness (Thannickal et al. 2014; Wells 2013).

Threespine stickleback ( *Gasterosteus aculeatus* ; hereafter ‘stickleback’) are often infected with *Schistocephalus solidus* , parasitic tapeworms that develop within the body cavity of infected hosts (Barber and Scharsack 2010). Stickleback are the intermediate hosts of *S. solidus* and, once the parasite reaches the infectious stage (~50 mg), infected stickleback display complex parasite‐induced behavioural modifications that facilitate transmission to the final bird hosts (Barber 2013). Stickleback anti‐ *S. solidus* responses partially occur via the development of fibrosis, which can limit the size of *S. solidus* (preventing parasites from reaching the infectious stage) and can even eliminate infections (Fuess et al. 2021; Weber et al. 2022). Although seemingly advantageous, stickleback that develop fibrosis must then bear the burden of this irreversible and costly immune response, which can include lower reproductive fitness and foraging rates (De Lisle and Bolnick 2021). Fibrosis can also alter stickleback locomotion, including aspects of the C‐start response (rapid, small‐scale movement that typically occurs in response to an immediate threat, such as a predation attempt; Matthews et al. 2023). However, it is not yet known how fibrosis influences larger‐scale movements—such as dispersal in a lake.

Our objective was to assess the extent to which fibrosis influences stickleback dispersal in nature. To investigate this, we conducted a mark‐recapture experiment in a lake in Alaska, where fibrosis was experimentally induced in a subset of fish. We found that fibrosis does not affect dispersal (estimated as distance captured from the release point). However, interesting trends, although not statistically significant, suggest that the timing of fibrosis development may affect dispersal, but additional work will be needed to provide support for these suggestive trends.

## 2 Materials and Methods

### 2.1 Study Location

The work for this project was conducted in June 2023 in Hope Lake (60.421467°, −151.187413°) on the Kenai Peninsula in Alaska. In 2019, stickleback were introduced into Hope Lake from four other populations as part of a series of stickleback introductions in Alaska (Hendry et al. 2024). We chose Hope Lake due to the low natural fibrosis rates in 2023 relative to other Kenai Peninsula lakes (Bolnick et al. 2024).

### 2.2 Capture, Mark and Release

**FIGURE 1:**
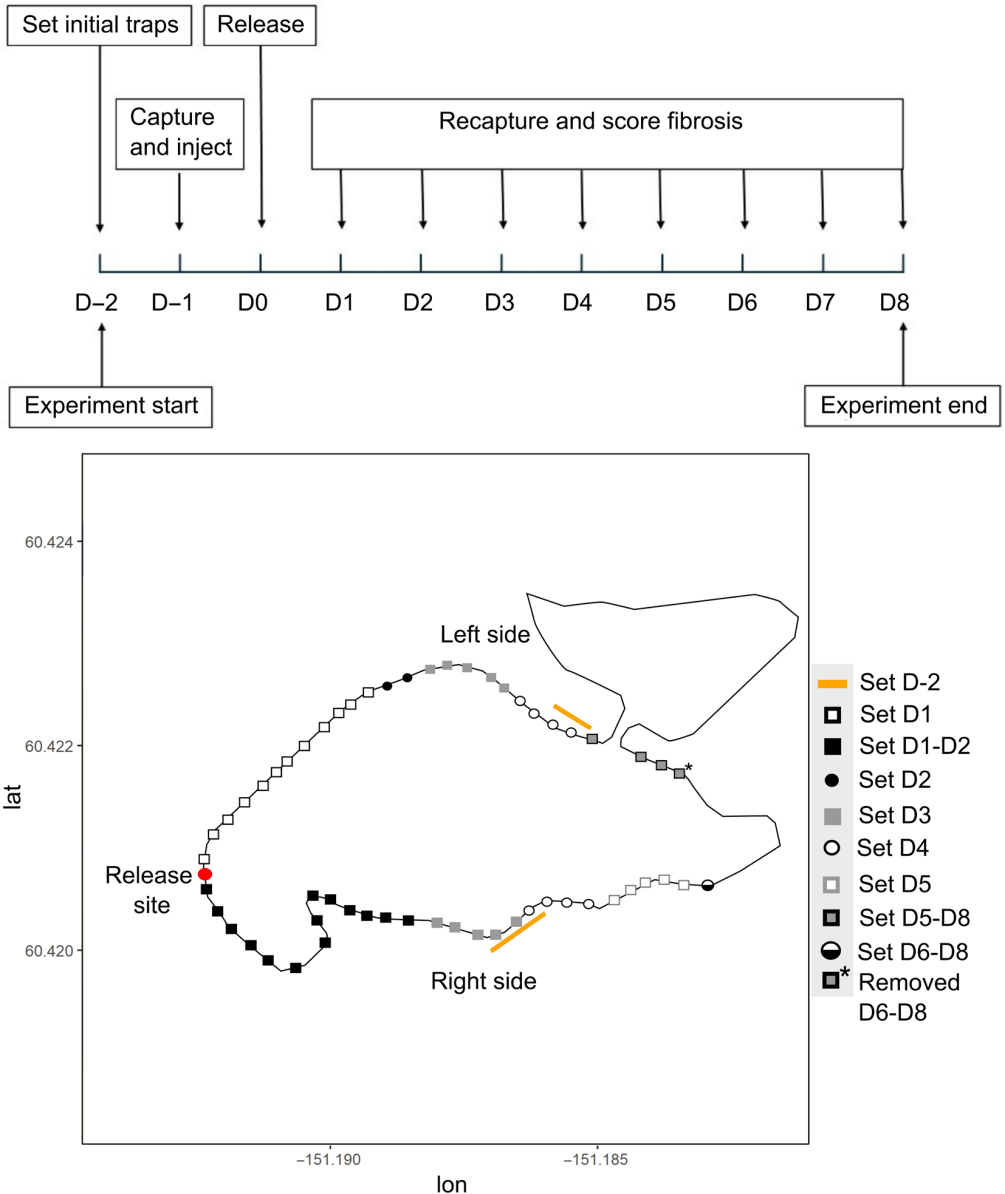
Experimental timeline and map of Hope Lake. On the map, the red circular point represents the release point, and the orange lines represent the stretch of shoreline where twenty traps were set (ten on either side) to capture stickleback for use in the experiment (this line was shifted latitudinally to avoid covering the other points). The other points represent trap locations set throughout the experiment. The colours and shapes of the data points indicate the day on which the traps were set; the traps that were added on a given day (abbreviated ‘D’) were set in the same location for all subsequent days of the experiment, except in one case where a trap was removed after only being set for a single day (indicated by the asterisk). The sides of the lake are labelled relative to the release site (left/right).

Minnow traps were set on June 18th at approximately midnight (D‐2 on Figure 1) and were checked at 07 h on June 19th, 2023. Traps were set midway up the lake on both sides. Fish captured on either side of the lake from our access points were kept separate during handling and processing (D‐1). We presumed all stickleback included in the experiment were adults based on body size, although it is possible that we included some sub‐adults because stickleback show substantial among‐population variation in adult body sizes (Reimchen, Steeves, and Bergstrom 2016).

Prior to injections, stickleback were anaesthetised using buffered MS‐222 (40–75 mg/L pH 7.4). For injections, individual fish were placed on kitchen sponges covered in wet paper towels, with a second piece of wet paper towel covering the eyes and gills. A subset of fish (*n* = 300) were injected intraperitoneally with 10 μL of alumvax phosphate (OZ Biosciences, San Diego, CA, USA; ‘alum’), a vaccine adjuvant that recruits immune cells to the injection site, causing strong innate immune responses (Kool, Fierens, and Lambrecht 2012) and inducing fibrosis in ray‐finned fishes (Vrtílek and Bolnick 2020). The fibrotic response induced by these alum injections does not differ among any of the stickleback populations introduced into Hope Lake (Bolnick et al. 2024). Another subset of fish (*n* = 300) were injected with 10 μL of phosphate buffered saline (EMD Milipore, Billerica, MA, USA), which does not induce fibrosis. We used Ultra‐Fine needle insulin syringes (capacity: 3/10 mL; length: 8 mm; gauge: 31 g; BD Bioscience, Franklin Lakes, NJ, USA) for the injections. A final subset of fish (*n* = 57) were anaesthetised but received no injections to ensure that the injections did not cause fibrosis. Fish had their dorsal spine, left pelvic spine, or right pelvic spine clipped to indicate if they received the alum, saline, or handling‐only treatment, respectively.

Alum injections occurred from approximately 09 to 19 h. Saline injections occurred from approximately 19 to 22 h. Saline injections occurred over a much shorter time because the saline syringes were filled in advance, which is not possible for alum because the alum solution separates and requires frequent mixing—although individuals in both treatment groups were held in captivity and handled during injections for approximately the same amount of time. Dorsal spine clips for the handling‐only control group took place from approximately 23 h to 01 h. Upon injection, the fish were immediately placed in a recovery bucket outfitted with a bubbler for at least 15 min where their swimming was monitored to ensure that they resumed normal behaviour. The fish were then transferred to larger coolers outfitted with bubblers, where they were held until they were released. Approximately 24 h following the first alum injections (D0), we released 223 fish that received alum injections, 263 fish that received saline injections and 50 fish that received no injection back into Hope Lake at one common introduction point.

### 2.3 Recapture and Fibrosis Scoring

Every day for the next 8 days (D1–D8), we set traps along both sides of the lake spaced approximately 20 m apart, starting from the introduction point. On the first day of recapturing (D1), we expected that fish would not move more than 200 m in 24 h and so we set the farthest traps at 200 m (Bolnick et al. 2009). If marked fish were found in the farthest trap, we added additional traps at 20 m increments on the subsequent days (Figure 1). On D5 no marked fish were captured in the two farthest traps on the left side, and so we removed the single farthest trap on the left side for the subsequent day so that we could add an additional trap on the right side. For the final 3 days (D6–D8), we were trapping close to 500 m from the introduction point on both the left and right sides of the lake (475 m on the left side and 505 m on the right side).

We set the first traps each morning of the experiment at approximately 9 h along the shoreline on one side of the lake (i.e. the left or right side looking outward from the introduction point). Midway through the day, we would set traps along the opposite shoreline. On both sides, we started by setting the traps that were the farthest from the introduction point (it took approximately 10–20 min to set all the traps). We started checking the traps approximately 3 h after the first traps were set (~12 h). Each day we would check the farthest traps from the release point until we captured five marked fish on both sides of the lake—those presumed to have dispersed the farthest. If we captured more than five fish, the ‘extra’ marked fish would be returned to the lake at the point of capture (*n* = 15); we returned these fish because fibrosis can take days to develop, and we wanted to maximise variation in fibrosis and dispersal distance for our analyses. We would then return to the shore and score fibrosis (see below), after which we would return to the lake and check the closest traps until we captured five additional marked fish per side—those presumed to have dispersed the least. To ensure that the time of day did not bias our results, we alternated daily which side of the lake was checked first (e.g. on day one we would set traps on the left side of the lake first, and then on day two we would set traps on the right side first). GPS coordinates were collected for each trap, and the distance between the common release point and the trap from which a given stickleback was sampled was used as a proxy of dispersal distance.

We euthanised the fish with a lethal dose of buffered MS‐222. Fish were dissected, and fibrosis was scored on a scale of 0–4 (Table 1) (Hund et al. 2022). Fish sex, mass (g), standard length (mm), and presence/absence of *S. solidus* were recorded. Carcasses were retained and stored in formalin and brought back to McGill University. These experiments were conducted in accordance with approved IACUC protocols at the University of Illinois Urbana‐Champaign (Protocol #: 21031), with permission of the State of Alaska Department of Fish and Game (Permit #: SF2023‐110).

**TABLE 1:** Definitions used to score fibrosis (Hund et al. 2022).

Fibrosis score	Definition
0	No fibrosis—visceral organs move around freely.
1	Mild fibrosis—visceral organs are slightly fused together but still move around relatively easily.
2	Moderate fibrosis—visceral organs are fused together, but are not fused to the body wall.
3	Moderate‐severe fibrosis—visceral organs are fused together and to the body wall. Skin does not tear when the fish is dissected.
4	Severe fibrosis—visceral organs are completely fused together and to the body wall. Skin tears when the fish is dissected.

### 2.4 Statistical Analysis

Statistical analyses were performed in R v 4.3.1 (R Core Team 2024). A Wilcoxon signed rank test confirmed that fibrosis did not differ between the injection control (saline injections) and the handling control (dorsal clips) groups (*W* = 398.50, *p* = 0.23), so the control and handling control fish were pooled to create a broader ‘control’ group variable for subsequent analyses. We also used a Wilcoxon signed rank test to confirm that alum injections successfully induced higher extents of fibrosis, and we calculated the mean fibrosis score (±SD) for alum‐injected and control group fish. We used a chi‐squared test to confirm no differences in recapture rates between the treatment and control groups (see ‘Results 3’).

To investigate the effects of fibrosis on dispersal, we started by calculating the median dispersal distance for fish with or without fibrosis. We also compared the median dispersal distance between alum‐injected and control‐group fish, but only for fibrotic fish. This comparison accounts for two considerations. First, fish that received alum injections might not have developed fibrosis by the time they were captured; for some stickleback genotypes, fibrosis can take ~10 days to develop (Hund et al. 2022), and our experiment ended approximately 9 days after we did the injections. Second, fish from the control group could have pre‐existing fibrosis not caused by our experimental treatment. This comparison therefore allows us to roughly compare induced fibrosis (in the alum treatment fish) to pre‐existing fibrosis (in the control group fish). We finally calculated the median dispersal distance between alum‐injected non‐fibrotic fish and control group non‐fibrotic fish. Differences between medians within these three groups were formally evaluated using Wilcoxon signed rank tests. Below, we present results from analyses conducted with the full dataset, but complementary analyses conducted using only fibrotic fish and only non‐fibrotic fish can be found in the (Tables S3–S7).

To investigate the effects of fibrosis on dispersal, we used a structural equation model (SEM) (Rosseel 2012). This approach is appropriate because of the hierarchical nature of our analyses: a treatment (i.e. alum) induces fibrosis, which may or may not induce a change in dispersal distance. Path analyses can be more effective than traditional linear mixed models for detecting the effects of alum‐induced fibrosis on behaviour (Matthews et al. 2023).

Before modelling, continuous predictors were scaled and centred, and one data point (a saline‐injected individual) was removed from the dataset owing to incomplete data collected during the dissections. We first built a base model, comprised of the direct effect of treatment on fibrosis and the direct effects of treatment and fibrosis on dispersal distance. Treatment was therefore considered to have an indirect effect on dispersal via effects on fibrosis. In a second model, in addition to the base model, we also included the direct effects of sex on fibrosis and dispersal distance, as well as mass (g) on dispersal distance. Although we also collected standard length, we only included mass (g) in this model because mass (g) and standard length (mm) were highly correlated (*r* = 0.88). In this second model, we also included the direct effects of maximum trap distance on fibrosis and maximum trap distance on dispersal distance. The former effect is important to consider because fibrosis can take a few days to develop, and so fibrosis could increase throughout the experiment. The latter effect is important because we set traps on both sides of the lake, and there could be a side bias, and, because we increased the number of traps during the experiment, the farthest distance the fish could be captured on D1 was not as far as on D8 (i.e. maximum trap distance covaries with the day of the experiment). Finally, although we also recorded the presence of *S. solidus* , prevalence was very low (3%; *n* = 4) and so we did not include *S. solidus* prevalence in the models. In summary, this second model included effects of treatment, fibrosis, sex, mass (g) and maximum trap distance (m). We compared the base model to the more complex model with AIC, and the more complex model was considered superior (ΔAIC = 53.07).

To corroborate the SEM findings, we constructed linear mixed models (LMM) (Bates et al. 2015). We constructed a base model with the independent effects of fibrosis, treatment, sex and mass (g) on dispersal distance (no interactions). We also constructed a second model with a four‐way interaction between fibrosis, treatment, sex and mass (g). In both models, we included maximum trap distance (m) as a random effect to account for side and day effects. The complex model with interactions was the best model (ΔAIC = 71.83); however, no interactions were statistically significant (Table S2), and so the interactions were removed to optimise model fit (Engqvist 2005). For effect sizes, we calculated part *R* ^2^ for each predictor in the final model using 1000 bootstrap iterations for 95% confidence interval estimation (Stoffel, Nakagawa, and Schielzeth 2020). No linear model assumptions were violated (Hartig 2022).

## 3 Results

We recaptured 23% (*n* = 123) of the fish that we released into Hope Lake. Within each treatment group, we recaptured 21% of the alum (*n* = 47), 25% of the saline (*n* = 66), and 20% of the no injection (*n* = 10) fish. Alum injection induced higher extents of fibrosis (*W* = 2343, *p* < 0.01) (mean fibrosis ±SD: alum‐injected fish = 1.28 ± 1.16; control‐group fish = 0.66 ± 0.97), and recapture rates did not differ between the treatment and control groups (*χ* ^2^ = 0.36, df = 1, *p* = 0.55).

**FIGURE 2:**
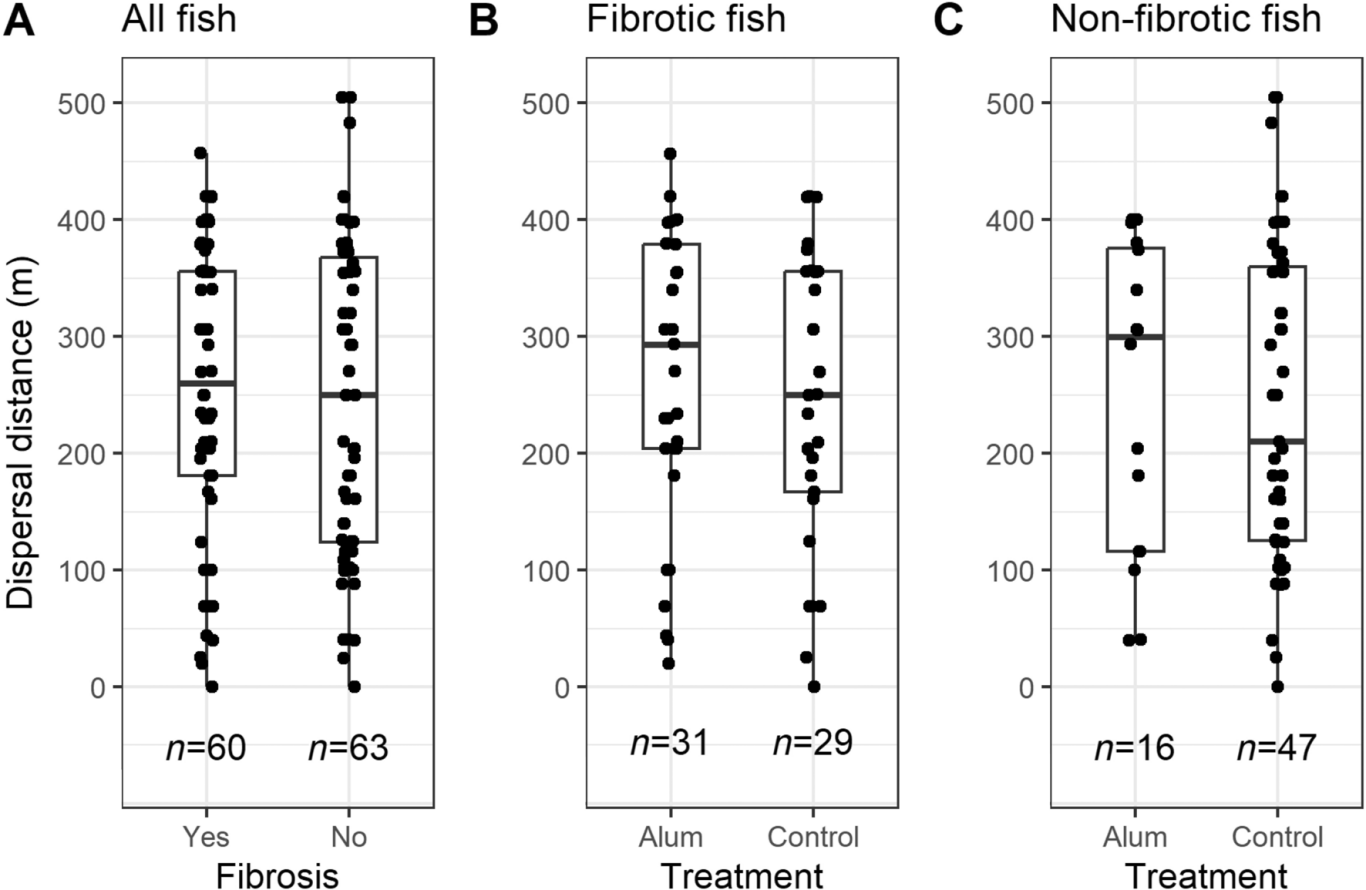
Although (A) fibrosis does not affect dispersal overall, non‐statistically significant trends suggest alum‐injected fish may disperse farther than control‐group fish when only considering (B) fibrotic fish (comparing induced fibrosis to long‐standing fibrosis) and (C) only non‐fibrotic fish (comparing induced, early‐stage fibrosis that is not yet visible to no fibrosis). The midline of the boxplot denotes the median, and the jittered data points represent individual fish. Sample sizes are displayed for each group. The differences presented within each panel are not statistically significant.

Only 24 h post release (D1), half of the captured fish had dispersed more than 100 m from the introduction point, and, by midway through the experiment (~ D4), over half the captured fish had dispersed more than 300 m (Table 2). Median dispersal distances did not significantly differ when comparing all fish (fibrotic vs. non‐fibrotic: *W* = 1932, *p* = 0.45), only fibrotic fish (alum vs. control: *W* = 491.5, *p* = 0.54) and only non‐fibrotic fish (alum vs. control: *W* = 392.5, *p* = 0.80). Nevertheless, irrespective of treatment, the median distance (and interquartile range; IQR) that fibrotic fish dispersed was 260 m (175 m) compared to 250 m (243.5 m) for fish without fibrosis (Figure 2A). Conversely, among only fibrotic fish, the median distance (IQR) that alum‐injected fish dispersed was 293 m (175 m), compared to 250 m (189 m) for control group fish (Figure 2B). This comparison was even more exaggerated between the non‐fibrotic alum‐injected fish, which moved a median distance (IQR) of 300 m (259.5 m), compared to the non‐fibrotic control‐group fish, which moved 210 m (234.5 m) (Figure 2C).

**TABLE 2:** The median distance (and interquartile range; IQR) at which stickleback were captured for each day of recapturing, along with the maximum possible distance that they could be captured. See Table S1 for trapping information specific to each side of the lake.

Day	*n*	Median capture distance (m)	IQR	Maximum trap distance (m)
D1	23	100	109.50	250
D2	12	181	77.50	293
D3	15	293	145.50	356
D4	19	320	119.50	398
D5	14	347	278.75	498
D6	21	398	150.00	505
D7	15	340	138.50	505
D8	4	356	56.00	505

**FIGURE 3:**
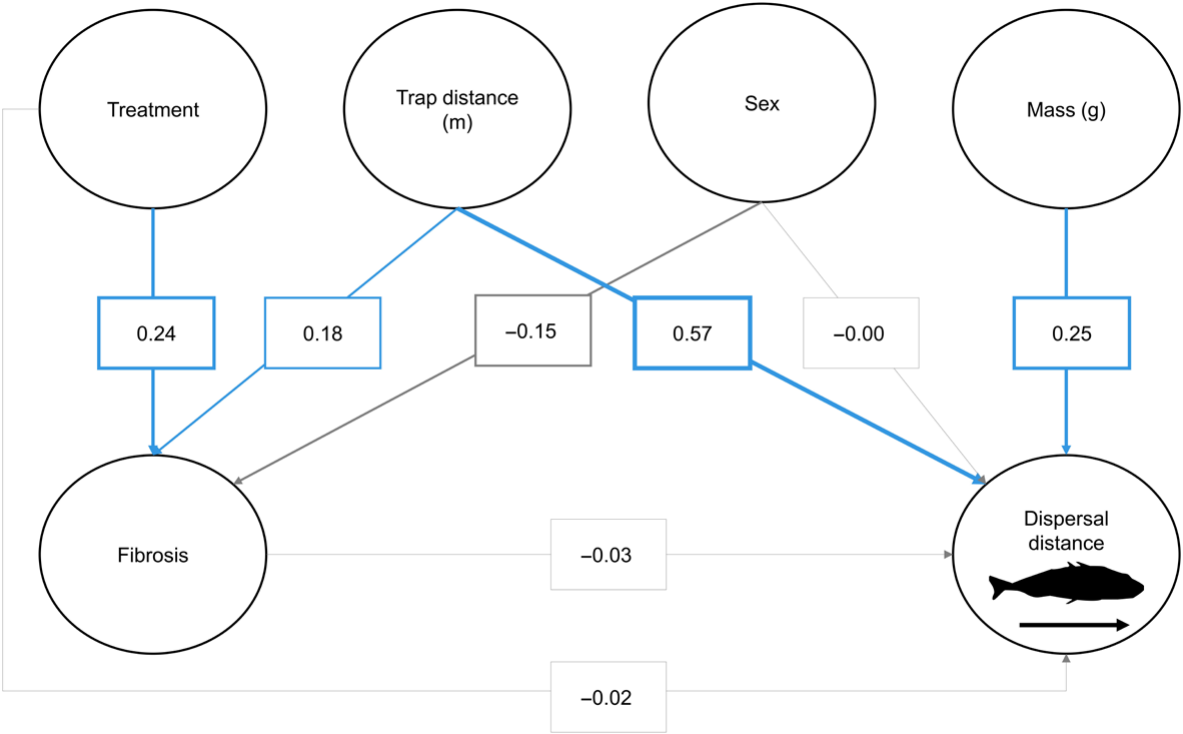
SEM results showing that fibrosis does not affect dispersal, although some other factors do. The blue lines indicate positive associations, the grey lines indicate negative associations, and the numeric values represent standardised path coefficients, which are also reflected in the width of the lines.

**FIGURE 4:**
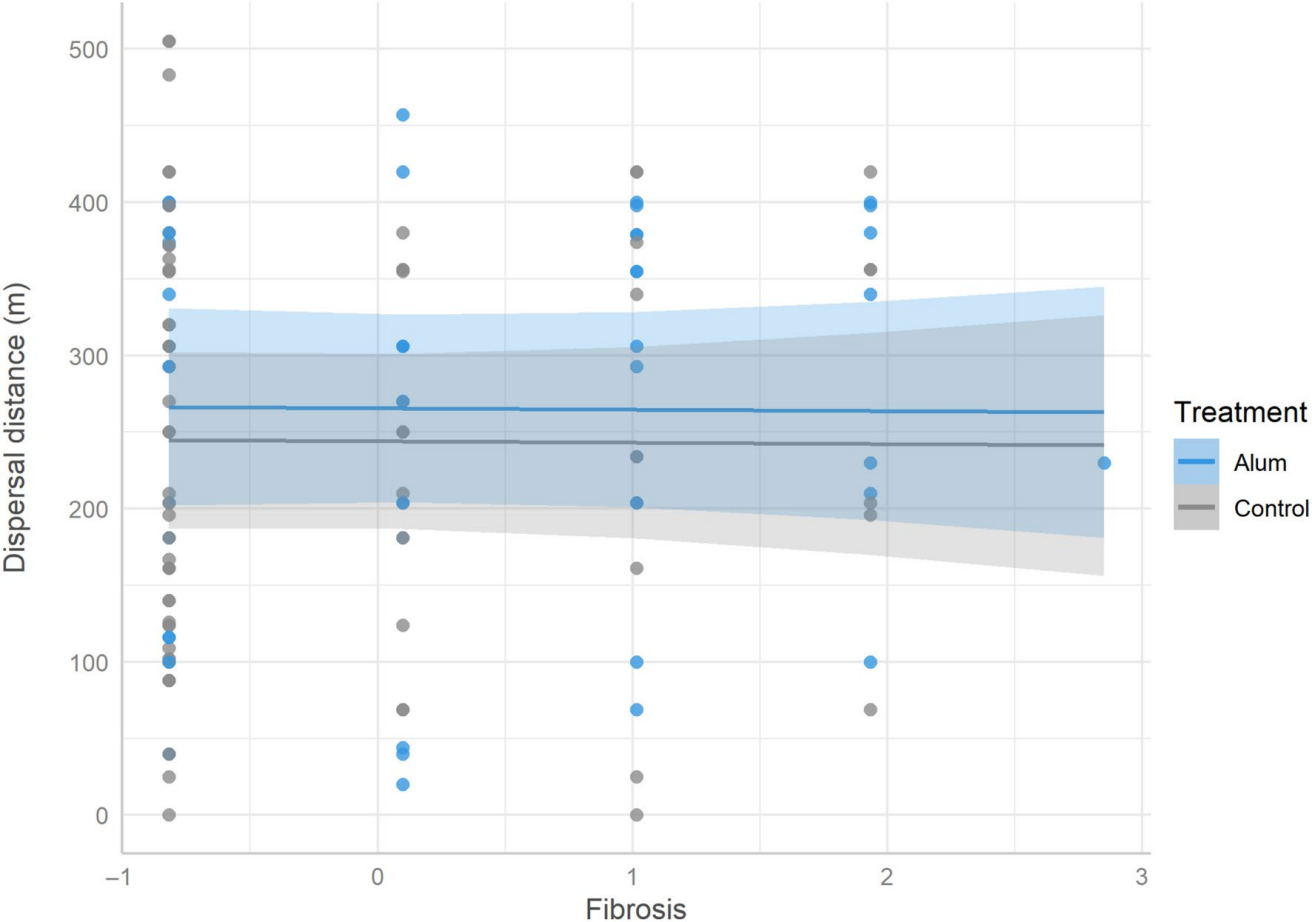
LMM results showing that fibrosis does not affect dispersal. The lines and ribbons are predicted by the model, and the data points are the raw data. Fibrosis is *z*‐score standardised, so values on the *x*‐axis do not correspond to the literal measured fibrosis scores.

With the SEM, we did not find an effect of fibrosis on dispersal (Table 3). Unsurprisingly, treatment had a direct effect increasing fibrosis, and maximum trap distance was significantly associated with dispersal distance, confirming the value in including these terms in the models (Figure 3; Table 3). Mass (g) and maximum trap distance (m) also significantly increased dispersal distance, whereas neither sex nor treatment were statistically significant (Figure 3; Table 3). Consistent with these SEM results, only mass (g) was significant in the LMMs and explained about 6% of the variance in dispersal (part *R* ^2^ [95% CI] = 0.06 [0.02–0.16]) (Table 4). The effect sizes for all other predictors were extremely low (part *R* ^2^ [95% CI]: treatment = 0.01 [0.00–0.11], fibrosis = 0.00 [0.00–0.10], sex = 0.00 [0.00–0.10]) (Figure 4).

**TABLE 3:** SEM regression results. Fibrosis does not affect dispersal, but maximum trap distance (m) and mass (g) do. Fibrosis is affected by treatment and maximum trap distance (m).

Response variable	Explanatory variable	Estimate	SE	*z*	*p*
Fibrosis	**Treatment**	**0.50**	**0.17**	**2.85**	**< 0.01**
	**Maximum trap distance (m)**	**0.19**	**0.09**	**2.17**	**0.03**
	Sex	−0.30	0.17	−1.76	0.08
Dispersal distance	Fibrosis	−3.75	10.08	−0.97	0.71
	Treatment	−4.58	20.06	−0.23	0.82
	**Maximum trap distance (m)**	**73.92**	**9.69**	**7.63**	**< 0.001**
	Sex	−0.40	19.15	−0.02	0.98
	**Mass (g)**	**32.41**	**9.38**	**3.46**	**< 0.01**

*Note:* Significant terms (*p* < 0.05) are bolded.

**TABLE 4:** Results from the linear mixed models. Mass (g) is the only statistically significant factor affecting dispersal.

	*χ* ^2^	df	*p*
Fibrosis	0.01	1	0.92
Treatment	1.08	1	0.30
Sex	0.06	1	0.80
**Mass (g)**	**11.82**	**1**	< **0.001**

*Note:* Significant terms (*p* < 0.05) are bolded.

## 4 Discussion

We conducted a mark‐recapture experiment in a lake in Alaska to assess how an inflammatory and tissue repair response—peritoneal fibrosis—influences stickleback dispersal. We found that fibrosis does not affect dispersal. However, trends lacking statistical significance suggest that dispersal may be affected by the process of fibrosis development, highlighting opportunities for future work.

Given our large sample size (*n* = 123), our experiment is compelling in demonstrating that stickleback dispersal is not affected by fibrosis. This result is unexpected, owing to the physical side effects of fibrosis (e.g. increased body stiffness) and because aspects of fish locomotion are altered by fibrosis in laboratory settings (Matthews et al. 2023). It is possible that dispersal is not affected by fibrosis in nature because stickleback are able to compensate for the physical impacts to tissues by altering aspects of their locomotion (as can sometimes be observed for animals with injuries; Hendry et al. 2022). However, fibrosis is also costly for stickleback in natural settings, negatively affecting foraging success and reproductive fitness (De Lisle and Bolnick 2021), suggesting that not all costs of fibrosis can be rectified through compensatory behaviours, even when the severity of fibrosis is similar (mean fibrosis in De Lisle and Bolnick 2021 = 0.89 ± 0.90 vs. mean fibrosis here (ignoring treatment) = 0.89 ± 1.09). One difference between this past study and ours, however, is that whereas they worked with established fibrosis from infection, we experimentally induced fibrosis and our study therefore likely comprises more early‐stage fibrosis. Additionally, that study had much higher *S. solidus* prevalence (35% and 7% vs. 3% here). In populations with higher *S. solidus* prevalence, behavioural changes—including to dispersal—could be owing to fibrosis, as well as effects of the parasite. These effects of the parasite could emerge owing to morphological changes (e.g. distended abdomens and altered gait) or direct manipulation by the parasite on host behaviour (Barber 2013). Nevertheless, our findings raise the question of what other ecological performance features are affected by fibrosis in nature, if any? These might include predator evasion, male nest construction, courtship dances, nest maintenance and egg care.

The effects of fibrosis on dispersal are possibly better reflected in the statistically non‐significant trends suggesting that alum‐injected fish may disperse farther than control‐group fish. Although additional work is necessary to understand if the trend is indicative of something biologically meaningful, the trend suggests that although fibrosis itself does not affect dispersal, side effects of innate immune (inflammation) activation might, particularly in the earliest stages (i.e. before fibrosis is even visible). Indeed, whereas the median dispersal distance only differed by 10 m for fibrotic fish versus non‐fibrotic fish, this difference increased to 43 m for alum‐injected versus control‐group fibrotic fish and 90 m for alum‐injected versus control‐group non‐fibrotic fish.

There are several reasons why fish experiencing recent innate immune activation could disperse farther than fish with long‐standing fibrosis or no fibrosis, and these mechanisms could be investigated in future research. First, the majority of energetic costs associated with fibrosis could occur in the earliest stages of the immune response when fibrosis is developing. This shift in energetic requirements would explain why fibrosis itself does not affect dispersal, but fibrosis development may. Second, fish might be motivated to disperse to find environmental conditions that alleviate symptoms of discomfort or facilitate the immune response (Rakus, Ronsmans, and Vanderplasschen 2017). Future work that specifically compares variation in dispersal throughout fibrosis development, along with traits that can be affected by early immune responses (e.g. physiological, behavioural, metabolic), could provide insight into the possible role of early‐stage immune activation on ecological processes.

One limitation to our study is that we could not continuously track stickleback, and capturing stickleback at various distances from the introduction point only serves as a ‘proxy’ for dispersal. It is possible that a fish could have dispersed farther than we estimated, such as if a stickleback dispersed completely around the lake (~1500 m) before being trapped. This limitation could be circumvented if work is conducted in study systems where the cumulative dispersal distance can be acquired (e.g. using PIT‐telemetry). Continuous tracking could also potentially account for fish that occupy deeper sections of the lake (we were unable to capture these individuals because we trapped along the shoreline). Similarly, half of the stickleback in our study dispersed more than 100 m after 24 h in the lake, and the lake is < 800 m following the longest shoreline. Effects of fibrosis may have emerged had we conducted this study in a larger lake.

In conclusion, our study shows that stickleback dispersal in nature is not influenced by fibrosis. This finding can serve as a starting point for future work investigating other ecological processes that could be affected by fibrosis. The trends in our data that are not statistically significant also point to a possible utility of the stickleback‐fibrosis system for improving our understanding of the ecological implications of innate immune activation. In showing that peritoneal fibrosis, a motility‐restricting biological process, does not affect dispersal in a natural system, our study ultimately improves our understanding of how immune responses are associated with ecological processes in nature.

## Author Contributions

**Alexis M. Heckley:** conceptualization (equal), formal analysis (lead), investigation (lead), methodology (equal), writing – original draft (lead), writing – review and editing (equal). **Daniel I. Bolnick:** conceptualization (equal), methodology (equal), supervision (equal), writing – review and editing (equal). **Francis Dinh:** investigation (supporting), writing – review and editing (supporting). **Andrew P. Hendry:** conceptualization (equal), methodology (equal), writing – review and editing (supporting). **Natalie C. Steinel:** conceptualization (equal), methodology (equal), resources (equal), supervision (equal), writing – review and editing (equal).

## Conflicts of Interest

The authors declare no conflicts of interest.

## Supporting information


Tables S1–S7.

## Data Availability

The data and code required to reproduce the results from this study are available on Dryad repository.
