# Supplementary material for: Does Motility‐Restricting Fibrosis Influence Dispersal? An Experiment in Nature With Threespine Stickleback
Source: Ecol Evol. 2024 Dec 12;14(12):e70697. doi: 10.1002/ece3.70697 (PMC11635176; doi:10.1002/ece3.70697)
Supplement: Supplementary file 1 — Tables S1–S7. [file ECE3-14-e70697-s001.docx]

**Supplementary materials for: Does motility-restricting fibrosis influence dispersal?** **An experiment in nature with threespine stickleback.**

Table S1. The median distance (and interquartile range; IQR) at which stickleback were captured on either side of the lake (left/right) for each day of recapturing, along with the maximum possible distance that they could be captured.

| Day | Capture side | n | Maximum trap distance (m) | Median capture distance (m) | IQR |
| --- | --- | --- | --- | --- | --- |
| D1 | Left | 11 | 250 | 167 | 182 |
|  | Right | 12 | 204 | 100 | 42 |
| D2 | Left | 8 | 293 | 189 | 186 |
|  | Right | 4 | 204 | 181 | 6 |
| D3 | Left | 5 | 356 | 293 | 232 |
|  | Right | 10 | 306 | 255 | 125 |
| D4 | Left | 9 | 398 | 315 | 256 |
|  | Right | 9 | 379 | 320 | 49 |
|  | Release point | 1 | 398 | 0 | NA |
| D5 | Left | 4 | 498 | 374 | 34 |
|  | Right | 9 | 483 | 139 | 291 |
|  | Release point | 1 | 498 | 0 | NA |
| D6 | Left | 11 | 498 | 398 | 50 |
|  | Right | 10 | 505 | 347 | 195 |
| D7 | Left | 4 | 475 | 398 | 16 |
|  | Right | 11 | 505 | 230 | 136 |
| D8 | Left | 4 | 475 | 356 | 56 |
|  | Right | 0 | 505 | NA | NA |

Table S2. Results from the linear mixed model with a four-way interaction between fibrosis, treatment, sex, and mass, with a type 3 sum of squares. No explanatory variables are statistically associated with dispersal when considered independently or in an interaction.

|  | χ^2^ | df | p |
| --- | --- | --- | --- |
| **Intercept** | **51.86** | **1** | **<0.001** |
| Fibrosis | 0.17 | 1 | 0.68 |
| Treatment | 0.06 | 1 | 0.81 |
| Sex | 0.15 | 1 | 0.70 |
| Mass (g) | 0.00 | 1 | 0.99 |
| Fibrosis : Treatment | 0.27 | 1 | 0.60 |
| Fibrosis : Sex | 0.00 | 1 | 0.95 |
| Treatment : Sex | 0.64 | 1 | 0.42 |
| Fibrosis : Mass (g) | 0.20 | 1 | 0.65 |
| Treatment : Mass (g) | 2.25 | 1 | 0.13 |
| Sex : Mass (g) | 0.44 | 1 | 0.51 |
| Fibrosis : Treatment : Sex | 0.02 | 1 | 0.88 |
| Fibrosis : Treatment : Mass (g) | 0.02 | 1 | 0.87 |
| Fibrosis : Sex : Mass (g) | 0.26 | 1 | 0.61 |
| Treatment : Sex : Mass (g) | 1.52 | 1 | 0.22 |
| Fibrosis : Treatment : Sex : Mass (g) | 1.02 | 1 | 0.31 |

**Analyses with the subset datasets**

To complement the results in the main-text, we ran additional analyses with (1) a subset dataset comprised of only fibrotic fish (Tables S3-S5), and (2) a subset dataset comprised of only non-fibrotic fish (Tables S6-S7). For the SEMs, we only used the fibrotic fish dataset, because the objective of using SEMs was to assess possible effects of treatment on dispersal via fibrosis, and this analysis was therefore not possible without variation in fibrosis. The LMMs were conducted with both subset datasets – the only difference to the main-text is that the fixed effect of fibrosis was removed for the non-fibrotic fish LMM.

Table S3. SEM regression results conducted with the fibrotic fish dataset. Significant terms (p < 0.05) are bolded. Maximum trap distance (m) and sex significantly affected fibrosis, and only maximum trap distance significantly affected dispersal.

| Response variable | Explanatory variable | Estimate | SE | z | p |
| --- | --- | --- | --- | --- | --- |
| Fibrosis | Treatment | 0.31 | 0.23 | 1.33 | 0.18 |
|  | **Maximum trap distance (m)** | **0.33** | **0.11** | **2.91** | **<0.01** |
|  | **Sex** | **-0.66** | **0.23** | **-2.86** | **<0.01** |
| Dispersal distance | Fibrosis | -7.53 | 16.17 | -0.47 | 0.64 |
|  | Treatment | 12.61 | 29.17 | 0.43 | 0.67 |
|  | **Maximum trap distance (m)** | **62.99** | **15.25** | **4.13** | **<0.001** |
|  | Sex | -0.88 | 30.73 | -0.03 | 0.98 |
|  | Mass (g) | 21.46 | 14.34 | 1.50 | 0.14 |

Table S4. Results from the linear mixed model conducted with the fibrotic fish dataset. Maximum trap distance (m) was included as a random effect in the model. None of the measured factors had a statistically significant effects on dispersal distance.

|  | χ^2^ | df | p |
| --- | --- | --- | --- |
| Fibrosis | 0.06 | 1 | 0.81 |
| Treatment | 2.34 | 1 | 0.13 |
| Sex | 0.00 | 1 | 0.97 |
| Mass (g) | 0.91 | 1 | 0.34 |

Table S5. R^2^ values with confidence intervals from the linear mixed model conducted with the fibrotic fish dataset. Effect sizes were consistently weak. Confidence intervals were calculated using 1000 bootstrap iterations.

|  | R^2^ | 95% CI |
| --- | --- | --- |
| Model | 0.04 | 0.01-0.21 |
| Treatment (control) | 0.03 | 0.00-0.19 |
| Mass (g) | 0.01 | 0.00-0.17 |
| Fibrosis | 0.00 | 0.00-0.17 |
| Sex (male) | 0.00 | 0.00-0.17 |

Table S6. Results from the linear mixed model conducted with the non-fibrotic fish dataset. Maximum trap distance (m) was included as a random effect in the model. Significant terms (p < 0.05) are bolded.

|  | χ^2^ | df | p |
| --- | --- | --- | --- |
| Treatment | 0.20 | 1 | 0.65 |
| Sex | 0.01 | 1 | 0.93 |
| **Mass (g)** | **8.73** | **1** | **<0.01** |

Table S7. R^2^ values with confidence intervals from the linear mixed model conducted with the non-fibrotic fish dataset. Effect sizes were consistently weak. Confidence intervals were calculated using 1000 bootstrap iterations.

|  | R^2^ | 95% CI |
| --- | --- | --- |
| Model | 0.10 | 0.02-0.28 |
| Mass (g) | 0.09 | 0.02-0.28 |
| Treatment (control) | 0.00 | 0.00-0.19 |
| Sex (male) | 0.00 | 0.00-0.18 |
